# Segmentation of small ground glass opacity pulmonary nodules based on Markov random field energy and Bayesian probability difference

**DOI:** 10.1186/s12938-020-00793-0

**Published:** 2020-06-17

**Authors:** Shaorong Zhang, Xiangmeng Chen, Zhibin Zhu, Bao Feng, Yehang Chen, Wansheng Long

**Affiliations:** 1 School of Electronic Engineering and Automation, Guilin University of Electronic Technology, Guilin, 541004 China; 2 School of Mathematics and Computational Science, Guilin University of Electronic Technology, Guilin, 541004 China; 3 School of Electronic Information and Automation, Guilin University of Aerospace Technology, Guilin, 541004 China; 4 The Department of Radiology, The Affiliated Jiangmen Hospital of Sun Yat-sen University, Jiangmen, 529000 China

**Keywords:** Small GGO pulmonary nodules, Image segmentation, Active contour model, MRF energy, Bayesian probability

## Abstract

**Background:**

Image segmentation is an important part of computer-aided diagnosis (CAD), the segmentation of small ground glass opacity (GGO) pulmonary nodules is beneficial for the early detection of lung cancer. For the segmentation of small GGO pulmonary nodules, an integrated active contour model based on Markov random field energy and Bayesian probability difference (IACM_MRFEBPD) is proposed in this paper.

**Methods:**

First, the Markov random field (MRF) is constructed on the computed tomography (CT) images, then the MRF energy is calculated. The MRF energy is used to construct the region term. It can not only enhance the contrast between pulmonary nodule and the background region, but also solve the problem of intensity inhomogeneity using local spatial correlation information between neighboring pixels in the image. Second, the Gaussian mixture model is used to establish the probability model of the image, and the model parameters are estimated by the expectation maximization (EM) algorithm. So the Bayesian posterior probability difference of each pixel can be calculated. The probability difference is used to construct the boundary detection term, which is 0 at the boundary. Therefore, the blurred boundary problem can be solved. Finally, under the framework of the level set, the integrated active contour model is constructed.

**Results:**

To verify the effectiveness of the proposed method, the public data of the lung image database consortium and image database resource initiative (LIDC-IDRI) and the clinical data of the Affiliated Jiangmen Hospital of Sun Yat-sen University are used to perform experiments, and the intersection over union (IOU) score is used to evaluate the segmentation methods. Compared with other methods, the proposed method achieves the best results with the highest average IOU of 0.7444, 0.7503, and 0.7450 for LIDC-IDRI test set, clinical test set, and all test sets, respectively.

**Conclusions:**

The experiment results show that the proposed method can segment various small GGO pulmonary nodules more accurately and robustly, which is helpful for the accurate evaluation of medical imaging.

## Background

According to statistics, lung cancer has become the most common malignant tumor in the world, and it is also currently known as the cancer with the highest mortality after diagnosis [1]. The small ground glass opacity (GGO) pulmonary nodules are an early manifestation of lung cancer. Compared with solid pulmonary nodules, GGO pulmonary nodules have the characteristics of smaller diameter, lower contrast with surrounding normal lung tissue, intensity inhomogeneity, blurred boundary and irregular shape, which are often missed by doctors. Therefore, the detection and diagnosis of GGO pulmonary nodules have always been the focus and difficulty of imaging studies [2]. GGO pulmonary nodules are likely to become malignant tumors. If detected early, diagnosed early, and treated early, it will help reduce the risk of cancer [3]. In addition, small GGO pulmonary nodules are small size, and it is difficult to perform accurate puncture treatment at early stage. Therefore, multiple examinations are needed to pay close attention to their changes and evaluate them by medical imaging. The precise segmentation of GGO pulmonary nodules provides an important basis for medical imaging evaluation and diagnosis, so it has important clinical values.

At present, the segmentation methods of GGO pulmonary nodules mainly include mathematical morphology [4–6], active contour model [7, 8], and deep learning [9, 10]. The mathematical morphology method is based on set theory, and uses the structural elements of a given morphology to eliminate specific objects in the image. Kostis et al. [11] used the morphological algorithm with fixed size structural elements to distinguish small pulmonary nodules from surrounding vascular structures. Diciotti et al. [12] used mathematical morphological operations of corrosion and expansion to refine the segmentation results of pulmonary nodules. The active contour model method drives the curve or surface to deform by minimizing the energy function, thereby the target boundary can be reached. Farag et al. [13] proposed a level set-based pulmonary nodule segmentation algorithm to achieve adaptive segmentation of pulmonary nodules. Keshani et al. [14] used SVM classifier and active contour model to segment pulmonary nodules. Nithila et al. [8] used active contour model and fuzzy C-means clustering to segment pulmonary nodules. Li et al. [15] proposed an active contour based on adaptive local region energy function to segment GGO nodules. In recent years, deep learning method has been used to segment pulmonary nodules. Ye et al. [16] proposed a deep learning computer artificial intelligence system for early identification of GGO nodules. Roy et al. [17] proposed a collaborative combination of deep learning and shape-driven level set for automatic and accurate segmentation of pulmonary nodules. Wang et al. [18] proposed a central focus convolution neural network to segment pulmonary nodules. However, deep learning requires a large amount of labeled data, and it is still difficult to obtain a large number of pulmonary nodule labeled data. Most of the existing mathematical morphology and active contour model methods assume that the spatial location of each pixel in the image is statistically independent [19], which ignores the spatial structure information between pixels.

Markov random field (MRF) uses a neighborhood system to describe the relationship between neighboring pixels, which can well model the spatial structure information between pixels. Because of its small volume and easily affected by other factors such as blood vessels, pleura and surrounding highlight tissue, the segmentation of small GGO pulmonary nodules is vulnerable to boundary leakage. Making full use of the spatial structure information between pixels will help solve this problem. It is worth noting that the MRF has been widely used in other fields such as prostate glands [20] and brain MR image segmentation [21]. But it is still rarely used in the segmentation of pulmonary nodules. Zhu et al. [22] proposed an MRF method based on simulated annealing algorithm (abbreviated as MRF_SA) for segmentation of GGO pulmonary nodules, which achieved good results. However, no comprehensive research has been conducted on various complex types of GGO pulmonary nodules. In addition, the blurred boundary problem is not considered in [22].

Based on the literature [22], we incorporate MRF energy into the region term of active contour model, and propose an integrated active contour model based on Markov random field energy and Bayesian probability difference (IACM_MRFEBPD). First, the K-means method is used to pre-segment the image to solve the sub-optimal problem of traditional MRF segmentation, which improves the segmentation efficiency. Based on this, MRF model is constructed. Labeling field and feature field are established, and MRF energy is calculated. Instead of the intensity information, the MRF energy is used for constructing the region term of the active contour model. The MRF prior of the labeling field and the Gaussian mixture model of the feature field are based on local statistical information of the image. So the intensity inhomogeneity can be solved. In addition, MRF energy can enhance the contrast of the pulmonary nodule and the background region. Therefore, the low contrast problem can be solved. Second, the probability model of GGO pulmonary nodules and surrounding background regions is constructed using the Gauss mixture model and the model parameters are estimated by the expectation maximization (EM) algorithm. Then, the Bayesian probability difference of each pixel is calculated and used as the boundary detection function of the active contour model. Probability difference is 0 at the boundary; thereby the blurred boundary problem can be effectively solved. Therefore, the proposed method can segment the small GGO pulmonary nodules with intensity inhomogeneity, low contrast and blurred boundary.

## Results

### Experimental data and evaluation indicator

To verify the effectiveness of the proposed method, we use the public data of the Lung Image Database Consortium and Image Database Resource Initiative (LIDC-IDRI) [23] and the clinical data of the Affiliated Jiangmen Hospital of Sun Yat-sen University to perform experiments. The public data contain 632 CT images, and the clinical data contain 32 CT images. So the experimental data total 664 CT images. The GGO lung nodules selected in all CT images are less than 15 mm in diameter, and most are less than 3 mm. To train the deep learning model, we divide all data into training and test sets. The training set contains 376 LIDC-IDRI CT images, and the test set contains 256 LIDC-IDRI and 32 clinical CT images. The identifier (ID) number of the LIDC-IDRI data is given in Table 4 (see “Appendix”), where XX–YY represents the YYth CT image of the XXth patient. Related parameters of CT images such as tube current and tube voltage can be easily found by the ID numbers.

The proposed method is compared with the other five methods, including literature [24] (abbreviated as LGDF), literature [25] (abbreviated as SFCM_LCM), literature [26] (abbreviated as LRBAC), literature [22] (abbreviated as MRF_SA), and U-net model based on deep learning framework. The result manually segmented by radiologist is used as the gold standard. The pulmonary nodules less than 3 mm are located by LIDC-IDRI, but no gold standard is given. So most CT images have no gold standard. To solve this problem, we use the segmentation results of two radiologists in the Affiliated Jiangmen Hospital of Sun Yat-sen University as the gold standard. First, the two radiologists separately performed the segmentation, and then the combined results of the two people were used as the final gold standard.

In this paper, the intersection over union (IOU) score is used for evaluation indicator. The following is the calculation formula for IOU:

1 $${\text{IOU}} = \frac{{|A_{m} \cap A_{a} |}}{{|A_{m} \cup A_{a} |}} = \frac{\text{TP}}{{({\text{TP}} + {\text{FP}} + {\text{FN}})}}$$

where $A_{m}$ is the region segmented by experienced radiologist, $A_{a}$ the region segmented by methods, TP the true-positive, FP the false-positive and FN the false-negative. The larger the IOU, the better the segmentation effect.

### Model parameter setting

**Fig. 1:**
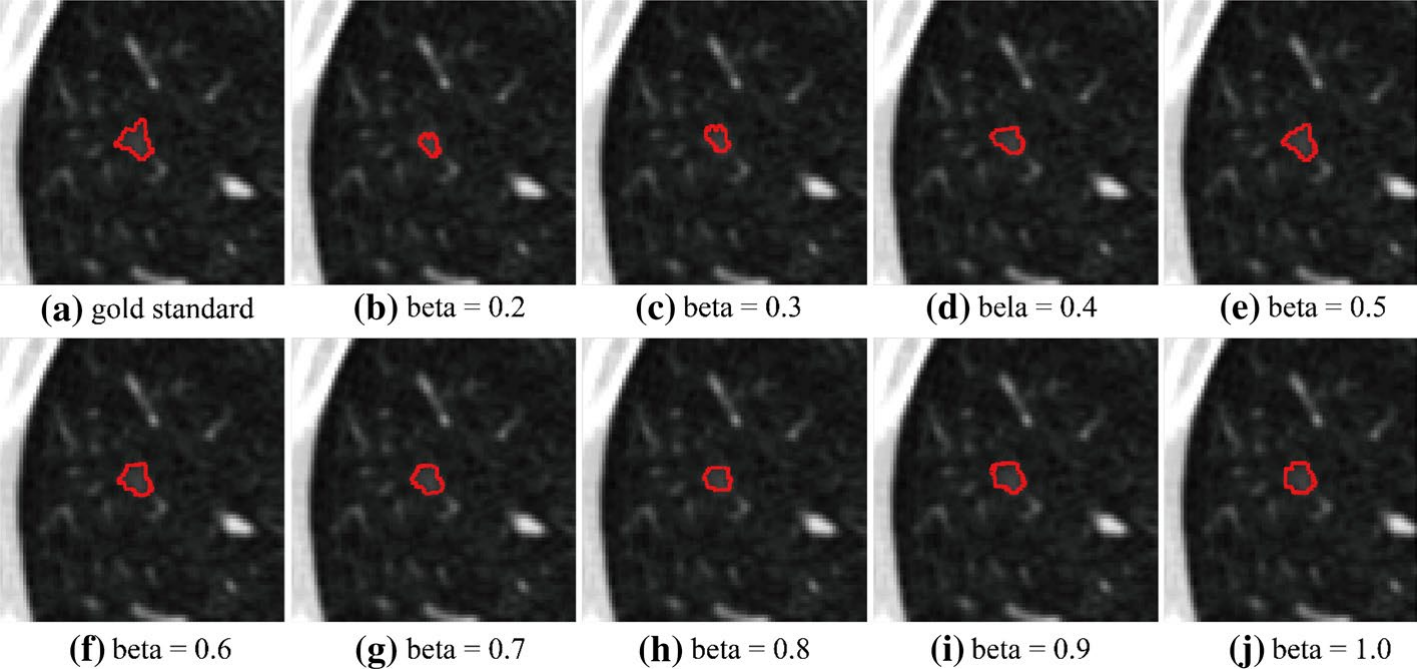
Segmented results with different beta values

**Fig. 2:**
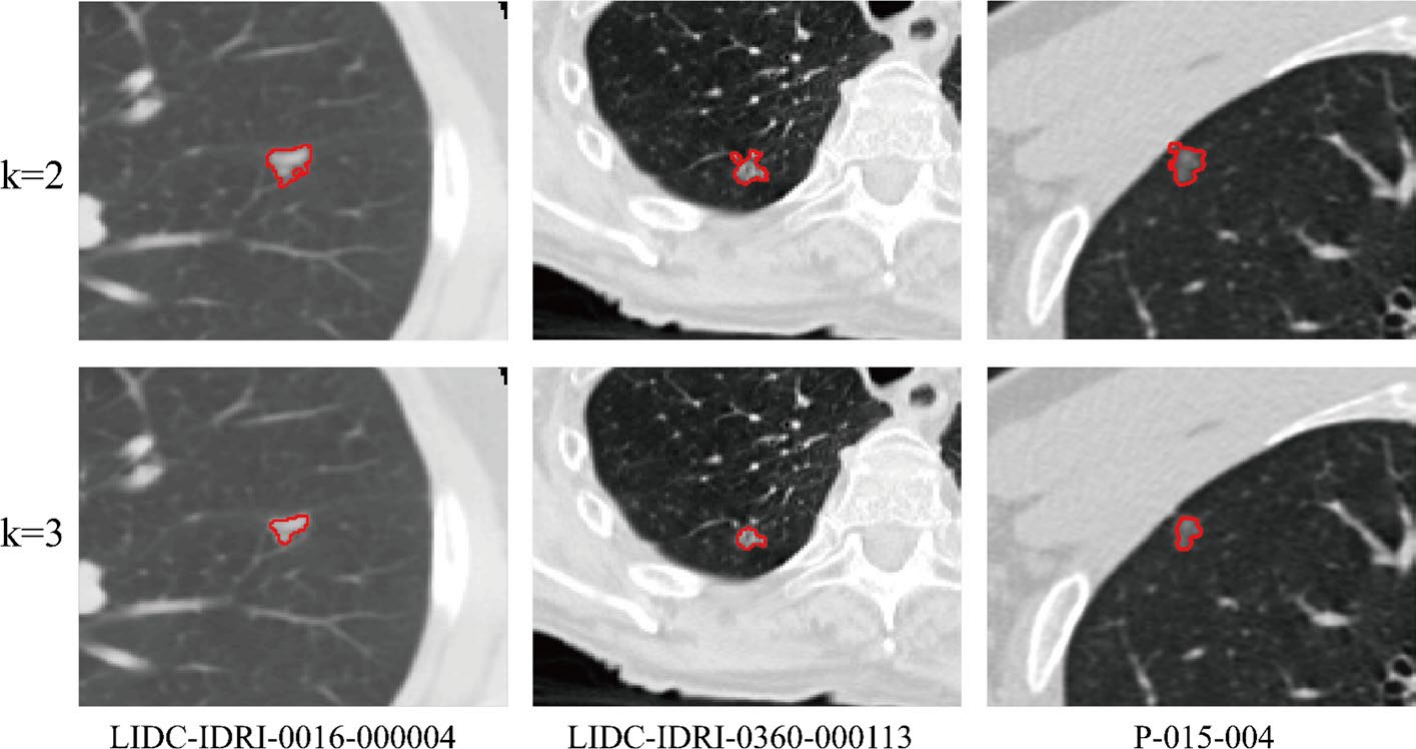
Segmented results with different *k* values

To set reasonable parameters for the proposed method, the comparative experiments are conducted on some important parameters. When calculating the MRF energy, the $\beta$ (beta) in formula (6) has a certain effect on the experimental results. Figure 1 shows the segmentation results of LIDC-IDRI-0380-000037 when beta changes from 0.1 to 1.0. As can be seen from Fig. 1, when *β* = 0.5, the segmentation effect is the best. After multiple trials and comparisons, we choose *β* = 0.5 for the segmentation of all CT images. When calculating the Bayesian posterior probability difference, *k* in formula (10) determines whether the GGO with vascular adhesion and pleural adhesion, and the GGO surrounded with highlight tissue can be correctly segmented. Figure 2 shows the segmentation results of *k* = 2 and *k* = 3 in these three cases. As can be seen from Fig. 2, to correctly segment these three types of GGO, the value of *k* should be set to 3. $\lambda_{1}$, $\lambda_{2}$, $\mu$ and $\nu$ in formula (15) are set to 1 as the suggest in [27]. The selection of other parameters will be discussed in “Methods” section. The parameter setting of the compared algorithm is consistent with the original literature.

**Fig. 3:**
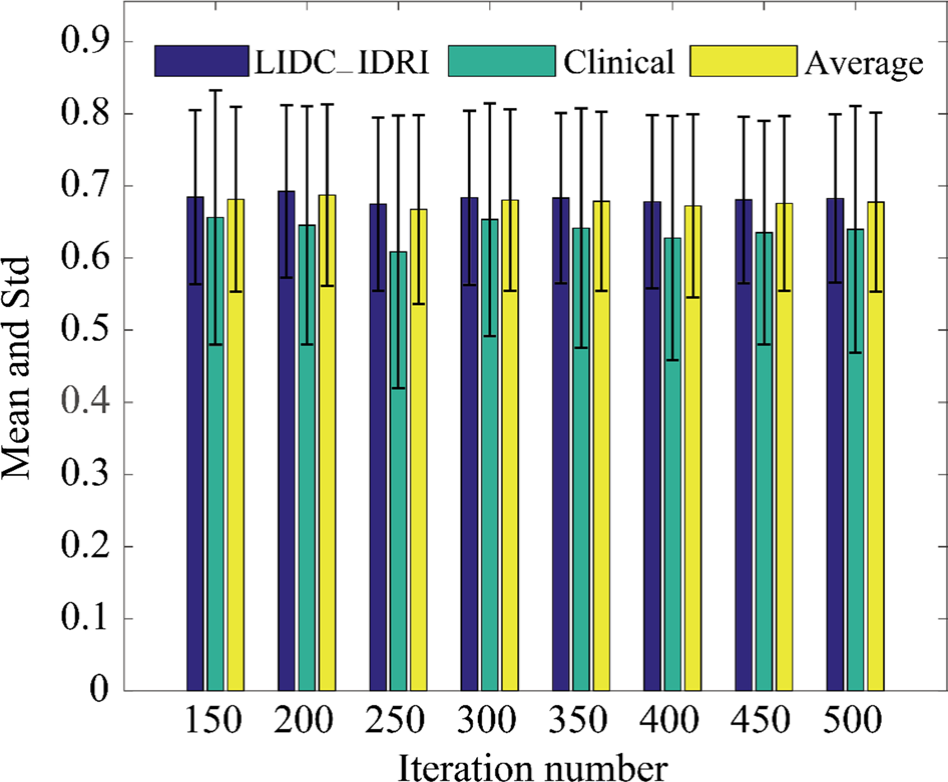
Average IOU with different iteration number

The parameter settings of U-net are as follows: batch size: 10, learning rate: 0.0001, loss function: binary cross-entropy. The segmentation result of the U-net method is related to the iteration number. Figure 3 shows the average IOU value of LIDC-IDRI test set, clinical test set, and the entire test set when the iteration number changes from 150 to 500. When the iteration number is 200, the average IOU value is the largest. So the iteration number of the U-net method is set to 200.

### Segmented results of LIDC-IDRI data

**Fig. 4:**
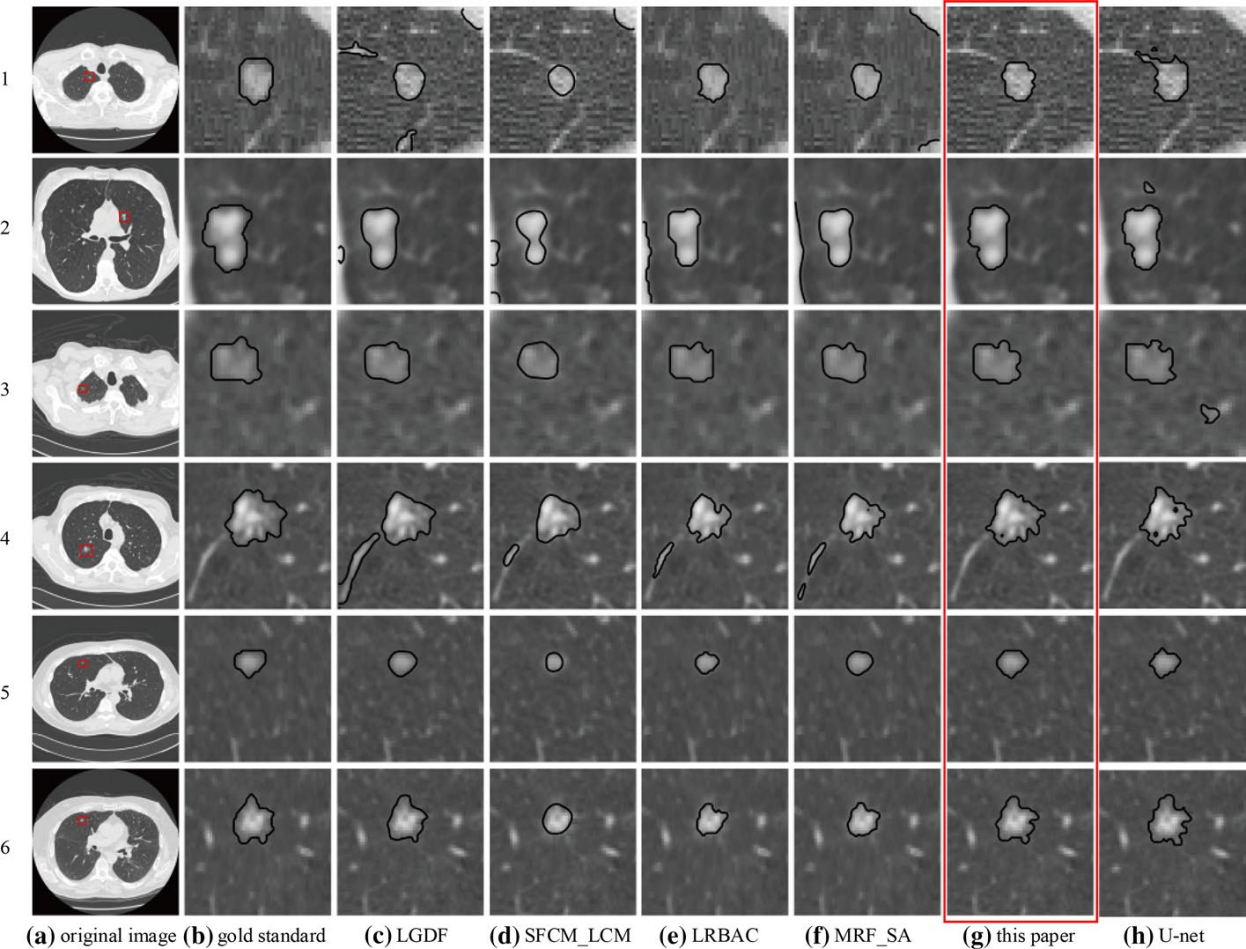
Segmented results of LIDC-IDRI data

Figure 4 shows the segmented results of 6 images with 6 methods. Figure 4a is the original image, and Fig. 4b is the segmented result by radiologist, which is the gold standard. Segmented results marked with red box are obtained by the proposed method. In the first image, the pulmonary nodule had mild vascular adhesion and the nodule was close to the pleura. The LGDF method (Fig. 4c) incorrectly segmented some of the blood vessels and a boundary leakage occurred at the pleura. Both the SFCM_LCM method (Fig. 4d) and the MRF_SA method (Fig. 4f) produced boundary leakage at the pleura. The U-net method (Fig. 4h) incorrectly segmented some of the blood vessels. Segmented results of the LRBAC method (Fig. 4e) are comparable to the proposed method. In the second image, the pulmonary nodule was relatively close to the pleura. Except that the proposed method can correctly segment, the U-net method mistakenly segmented some lung parenchyma and other algorithms produced the boundary leakage at the pleura. In the third image, the pulmonary nodule was relatively isolated but intensity inhomogeneity, and the shape was irregular. The SFCM_LCM method cannot correctly identify the shape of the pulmonary nodule; other methods worked comparable intuitively, but the U-net method mistakenly segmented some lung parenchyma. In the fourth image, the shape of the pulmonary nodule was extremely irregular, and with vascular adhesions. The proposed method and U-net correctly segmented, while other methods segmented some blood vessels. At the same time, other methods produced different degrees of under-segmentation due to blurred boundary. In the fifth image, the pulmonary nodule was relatively small, and with low contrast. The proposed method achieved better segmentation effect, while other methods produced under-segmentation, and the SFCM_LCM method was more serious. In the sixth image, boundary of the pulmonary nodule was blurred, and the boundary over-band was relatively large. In addition, the shape was irregular. The proposed method achieved a better segmentation effect and the U-net produced a certain over-segmentation, while other methods produced different degrees of under-segmentation, and the segmented boundary shrunk toward the interior of the pulmonary nodule.

In summary, the segmented boundary of the SFCM_LCM method is severely inwardly contracted and the shape of the pulmonary nodule cannot be identified. The proposed method has achieved better segmentation results, followed by the LRBAC method.

To better evaluate the performance of various methods, Table 1 gives the IOU scores for 6 LIDC-IDRI CT images in Fig. 4 and the average IOU for LIDC-IDRI test set. In addition, Table 1 shows the ID numbers of 6 CT images in Fig. 4. The optimal IOU score is highlighted in boldface. It can be seen from table that the proposed method achieved the best results, followed by LGDF. The effect of SFCM_LCM is the worst; the main reason is that there exists serious under-segmentation.

**Table 1:** IOU scores of LIDC-IDRI data

	CT image	LGDF	SFCM_LCM	LRBAC	MRF_SA	This paper	U-net
1	LIDC-IDRI-0759-000099	*0.7882*	0.5176	0.6941	0.7412	0.7412	0.6589
2	LIDC-IDRI-0294-000127	0.8289	0.4807	0.6558	0.6795	0.8373	*0.8567*
3	LIDC-IDRI-0743-000132	*0.9153*	0.5776	0.7931	0.8632	0.839	0.6348
4	LIDC-IDRI-0743-000201	*0.8678*	0.306	0.4181	0.6401	0.8021	0.7633
5	LIDC-IDRI-0400-000075	0.8841	0.5077	0.6769	0.8507	*0.9104*	0.7159
6	LIDC-IDRI-0375-000033	0.8343	0.3893	0.4765	0.6443	*0.8627*	0.715
Mean ± Std	All LIDC-IDRI test set	0.7217	0.4849	0.5692	0.6556	*0.7444*	0.6926

### Segmented results of clinical data

**Fig. 5:**
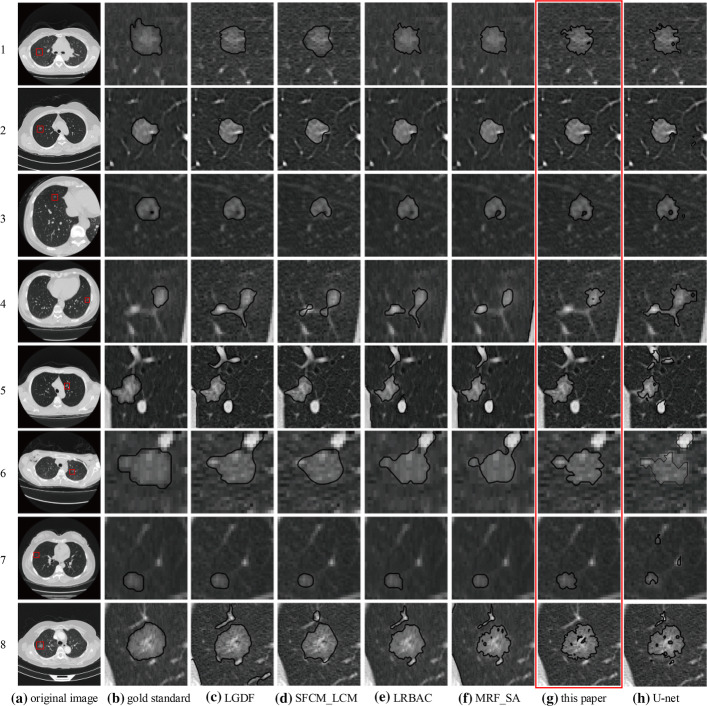
Segmented results of clinical data

Figure 5 shows the segmented results of 8 images with 6 methods. Figure 5a is the original image, and Fig. 5b is the segmented result by radiologist. Segmented results marked with red box are obtained by the proposed method. In the first three images, the pulmonary nodules were isolated, and with low contrast. Intuitively, all methods achieved the same effect, but the SFCM_LCM method produced a certain boundary leakage, and the segmented boundary shrunk inward. In the fourth image, blood vessel adhesion around the pulmonary nodule was relatively serious. The proposed method achieved good results, but other methods produced boundary leakage. In the fifth image, the pulmonary nodule was heavily adhered to the pleura and surrounded by blood vessels and other highlight tissues. Except for the proposed method, other methods all produced boundary leakage. In addition, the U-net produced serious under-segmentation. In the sixth image, the pulmonary nodule was close to the surrounding highlight tissue. Except for the SFCM_LCM method and the proposed method, other methods all produced boundary leakage, but the segmented boundary of SFCM_LCM method shrunk to the internal of the pulmonary nodule. In the seventh image, the pulmonary nodule was isolated, and with low contrast and small size. Except for the U-net method, other methods can segment the pulmonary nodule, but the segmented boundaries had different degrees of leakage, and the segmented boundary shrunk to the inside of the pulmonary nodule. In the eighth image, the pulmonary nodule was relatively large, but with intensity inhomogeneity, and there are many dark spots inside, which may be caused by bubbles or necrotic tissue. Furthermore, there are blood vessels adjacent to the nodules but not connected. Except for our method, other methods produced boundary leakage, and surrounding blood vessels were segmented. It is worth noting that the two methods based on MRF (MRF_SA and the proposed method) can correctly identify and segment the dark spots in the pulmonary nodule. It may be that the MRF effectively utilizes the spatial structure information of the image.

In the segmentation results of these 8 clinical CT images, the U-net effect is not good, with serious boundary leakage and under-segmentation.

Table 2 shows the IOU scores of 8 clinical CT images in Fig. 5 and the average IOU for clinical test set. It can be seen that the proposed method achieved better segmentation results with the highest average IOU, followed by LGDF. Although the proposed method has no obvious advantages in the segmentation results of a single CT image, it is stable in the segmentation results of multiple CT images. The IOU scores of other methods are very high for some CT images, but are very low in some case. In summary, the proposed method has good robustness.

**Table 2:** IOU scores of clinical data

	CT image	LGDF	SFCM_LCM	LRBAC	MRF_SA	This paper	U-net
1	P-001-001	0.7537	0.6784	0.7753	0.7453	0.5874	*0.8078*
2	P-002-025	*0.9253*	0.8375	0.9167	0.888	0.9205	0.7917
3	P-003-122	*0.8661*	0.6546	0.8314	0.8367	0.8392	0.8007
4	P-004-072	0.3525	0.6267	0.4153	0.3136	*0.6379*	0.2582
5	P-005-035	*0.7993*	*0.7993*	0.3152	0.3316	0.7964	0.3991
6	P-006-024	0.6705	*0.7703*	0.6627	0.6061	0.6403	0.6429
7	P-007-001	*0.9048*	0.7561	*0.9048*	*0.9048*	0.7619	0.375
8	P-008-029	*0.8623*	0.8136	0.7412	0.5339	0.7221	0.7706
Mean ± Std	All clinical test set	0.707	0.6057	0.5602	0.6456	*0.7503*	0.6453

Table 3 shows the average IOU of LIDC-IDRI data, clinical data, and all test sets. The proposed methods all achieved the highest average IOU, followed by LGDF.

**Table 3:** Average IOU scores of LIDC_IDRI test set, clinical test set and all test sets

	CT image	LGDF	SFCM_LCM	LRBAC	MRF_SA	This paper	U-net
1	LIDC_IDRI test set	0.7217	0.4849	0.5692	0.6556	*0.7444*	0.6926
2	Clinical test set	0.707	0.6057	0.5602	0.6456	*0.7503*	0.6453
3	All test set	0.7201	0.4983	0.5682	0.6545	*0.745*	0.6873

## Discussion

Based on the above-segmented results of LIDC data and clinical data, the proposed method has achieved better segmented results. In the segmentation experiments of clinical data, all methods have different degrees of boundary leakage, which are due to the influence of various factors such as blood vessels, pleura, and surrounding highlight tissue, etc. Therefore, the segmentation of small GGO pulmonary nodules is still very challenging.

LGDF method [24] uses Gaussian distributions with different mean and variance to describe local intensity information of the image, and uses truncated Gaussian kernel to define local attributes. The mean and variance of local intensity are considered as a function of spatial variation to deal with intensity inhomogeneity and spatially varying noise. However, the performance of the LGDF method depends more on the value of the local window size. When the value is not appropriate, the kernel function cannot be significantly reduced to zero. So it will cause boundary leakage. This phenomenon is particularly evident in the experiments of clinical data. In addition, the low contrast and blurred boundary are also the cause of the boundary leakage.

SFCM_LCM method [25] uses a spatially constrained fuzzy clustering method for the initialization and parameter control of the level set function, which promotes the evolution of the level set and improves the robustness of segmentation. At the same time, a local regular term is introduced to solve the problem of intensity inhomogeneity. However, the fuzzy nature of SFCM_LCM makes it impossible to correctly recognize the shape of pulmonary nodules. Therefore, for irregularly shaped pulmonary nodules, SFCM_LCM segmentation results are relatively poor.

LRBAC method [26] is a very effective local segmentation method, which increases the segmentation ability of intensity inhomogeneity images by introducing local statistical information. However, to achieve narrow-band control, the level set function in LRBAC needs to be re-initialized as a symbol distance function every few iterations during the process of model solving, and the calculation cost is high. At the same time, errors in the re-initialization process will cause the narrow-band control to be unstable, which affects the stability and robustness of the segmentation results. In addition, experiments have found that the segmentation results of LRBAC are sensitive to the selection of the local domain radius. If the local radius is too small, the segmentation may be insufficient, and if the local radius is too large, the segmentation may be excessive. In the actual pulmonary nodule segmentation, it is difficult to set a separate local radius for each segmentation. Finally, it should be noted that, compared with the global segmentation method, the local segmentation method is usually more sensitive to the initial contour. Therefore, the initial contour greatly affects the segmentation results of LRBAC.

Compared with MRF_SA method [22] and other MRF-based methods, the proposed method has two differences. First, the initial segmentation is helpful for the optimization of the MRF, and prevents the calculation from being stopped if it falls into a local minimum. Second, the MRF energy is calculated only once, and the MRF energy of each pixel no longer changes during the evolution of the contour. Unlike other MRF methods, this paper aims to use MRF energy to enhance the contrast of pulmonary nodules and background regions, rather than calculating the optimal solution of MRF in the framework of active contour models [21].

There are three main reasons why the proposed method has achieved good segmentation results. First, Markov’s prior is equivalent to performing a probabilistic morphological closing operation on the image, making spatially adjacent pixels more inclined to the same region, reducing the possibility of suspicious boundaries caused by noise and other tissues, such as blood vessels. Second, MRF energy calculation makes full use of the spatial structure information between pixels, which can enhance the contrast between pulmonary nodules and the background regions. So the problem of low contrast can be solved. At the same time, it is assumed that each pixel in the image conforms to a Gaussian mixture distribution when modeling the MRF feature field and this will make full use of local statistical information, which is helpful to solve the problem of intensity inhomogeneity. Third, the Bayesian probability difference is used for constructing boundary detection term. Probability difference is 0 at the segmentation boundary, which can well solve the blurred boundary problem. So the proposed method can deal with GGO pulmonary nodules with low contrast, intensity inhomogeneity and blurred boundary.

Finally, based on Bayesian posterior probability and initial segmentation by K-means method, the initial contour of the curve evolution can be obtained. Since the initial contour is located near the target boundary of image segmentation, the curve evolution can obtain the global minimum energy, which improves the stability and robustness of the proposed method. In the future work, we will continue to optimize the solution of the proposed model [28, 29].

## Conclusion

An integrated active contour model based on MRF energy and Bayesian probability difference is proposed in this paper. First, the MRF is constructed on the CT images and MRF energy is calculated. Instead of the intensity information, the MRF energy is used for constructing the region term. Second, the Gaussian mixture model is used to establish the probability model of pulmonary nodule image and the Bayesian posterior probability difference of each pixel is calculated. Next, the probability difference is used for constructing the boundary detection term. Finally, under the framework of the level set, the integrated active contour model is constructed. The experimental results of LIDC-IDRI data and clinical data show that the proposed method can segment various types of GGO pulmonary nodules more accurately and robustly than other methods. However, the proposed method does not specifically test for a certain type of GGO pulmonary nodules (such as GGO pulmonary nodules with different degrees of vascular adhesion) with a large number of samples, the robustness, stability and reliability of the proposed method need to be further verified. In the future work, when segmenting GGO pulmonary nodules with different degrees of vascular adhesion, we can combine MRF with shape information to accurately segment pulmonary nodules and blood vessels.

## Methods

**Fig. 6:**
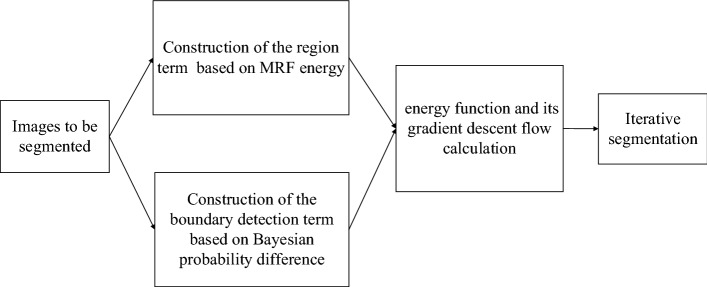
Image segmentation process

Assuming that $C$ is a closed contour curve, the image region $\varOmega$ is divided into region $\varOmega_{ 1}$ and $\varOmega_{ 2}$ by the curve, where $\varOmega_{ 1}$ represents the interior of the curve and $\varOmega_{ 2}$ represents the exterior of the curve. MRF energy instead of intensity information is used for constructing region term, which drives the curve to move to the target boundary. Bayesian probability difference instead of gradient information is used for constructing boundary detection term. The probability difference is 0 at the boundary, which stops the curve from evolving. Therefore, the energy function of the integrated active contour model can be defined as

2 $$E (\phi ,C )= \lambda E_{R} (\phi ) { + }\mu E_{E} (C )$$

where $E_{R} (\phi )$ is the region term, $E_{E} (C )$ is the boundary detection term, $\mu$ and $\lambda$ are two parameters that control the region term and the boundary detection term, respectively, and $\phi$ is the level set function. Figure 6 shows the image segmentation process of the integrated active contour model. The construction of region term and boundary detection term will be discussed later.

### Construction of the region term based on MRF energy

Traditional MRF is solved by multiple iterations, which is easy to fall into local optimum solution during the iterative process. The initial segmentation of the image can make the initial boundary near the real boundary, which improves the efficiency and stability of optimization solution, and effectively avoids the local optimal situation. Therefore, the initial segmentation can improve the accuracy and robustness of segmentation. In this paper, the K-Means algorithm is used to perform the initial segmentation of the original image, and then the MRF is constructed.

The image can be regarded as a two-dimensional MRF, whose pixel values are only related to its neighboring pixels. Assuming that the image size is $M*N$, the set of pixels in the image is represented by $S$, and $S = \{ s(i,j)|1 \le i \le M,1 \le j \le N\}$. Each element $s$ in the set $S$ is represented by neighborhood system. The potential clique ${\text{c}}$ is a subset of $S$, in which ${\text{c}}$ can be expressed as single-point potential clique, double-point potential clique and three-point potential clique, etc. As the order of the neighborhood system increases, the number of potential cliques will increase rapidly. Next, the MRF energy will be calculated using the neighborhood system and potential energy function.

First, constructing an MRF requires the construction of two fields, that is, labeling field and feature field. The labeling field is used to classify the image to be segmented, and the feature field is used to perform feature analysis on the classified region. The labeling field is represented by $X$, and $X = \{ x_{s} ,s \in S\}$. The labeling field value of any pixel is represented by $x_{s}$. The labeling space is represented by $\varLambda = \{ 1,2, \ldots ,L\}$, which divides the image into $L$ regions and $\varLambda$ is the set of random variables $x_{s}$, so $x_{s} \in \varLambda$ [22]. The feature field (or observation field) is represented by $Y$, and $Y = \{ y_{s} ,s \in S\}$. The feature field value of any pixel is represented by $y_{s}$. According to the Bayesian theory, the maximum posteriori probability can be expressed as

3 $$\hat{X} = \arg \mathop {\hbox{max} }\limits_{x} P(X|Y) = \arg \mathop {\hbox{max} }\limits_{x} \frac{P(X)P(Y|X)}{P(Y)}$$

where $Y$ is the observed image, so it can be regarded as a constant, which has no effect on maximizing the posterior probability. Therefore, the above formula can be written as follows:

4 $$\hat{X} = \arg \mathop {\hbox{max} }\limits_{x} P(X|Y) \propto \arg \mathop {\hbox{max} }\limits_{x} P(X)P(Y|X).$$

$P(X)$ is called segmentation model, which is modeled as MRF prior. It will be seen that $P(X)$ is a Gibbs distribution. Conditional probability $P(Y|X)$ is a data model, usually a Gaussian distribution; that is to say, the distribution of pixel value obeys Gaussian distribution after given label category [22]. To better fit the distribution of the pixel values, a mixed Gauss distribution is used for $P(Y|X)$ in this paper. In the following, we will model the labeling field (segmentation model $P(X)$) and the feature field (data model $P(Y|X)$), respectively.

By Hammersley–Clifford theorem, the prior probability $P(X)$ can be obtained [30], that is

5 $$P(X) = \prod\limits_{s \in S} {P(x_{s} )} = \prod\limits_{s \in S} {\frac{{\exp \left[ { - \sum\limits_{{{\text{c}} \in C}} {V_{\text{c}} (x_{s} )} } \right]}}{{\sum\limits_{{x_{s} = 1}}^{L} {\exp \left[ { - \sum\limits_{{{\text{c}} \in C}} {V_{\text{c}} (x_{s} )} } \right]} }}} ,$$

where $V_{\text{c}} (x_{s} )$ is the potential function of the potential clique ${\text{c}}$ containing $x_{s}$, and all potential clique set is represented by $C$. There are many ways to model $V_{\text{c}} (x_{s} )$ (labeling field modeling), such as Ising model, Potts model, MLL model, etc. In this paper, we use Potts model, which is consistent with literature [22]. The Potts model only considers the binary potential function, which is defined as

6 $$V_{\text{c}} (x_{s} ) = V_{2} (x_{i} ,x_{j} ) = \left\{ {\begin{array}{*{20}c} {0\quad x_{i} = x_{j} } \\ {\beta \quad x_{i} \ne x_{j} } \\ \end{array} } \right..$$

Next, the second-order neighborhood system is used and the corresponding potential cliques are two-point. $\beta$ denotes the parameter of the two-point potential clique, usually between 0.5 and 1.0. In our experiment, $\beta$ is set to 0.5.

Assuming that each pixel of the image obeys the independent and identical distribution, also obeys the Gauss mixture distribution. The conditional distribution of the feature field under a given labeling field is given as follows:

7 $$P(Y|X) = \prod\limits_{s \in S} {P(y_{S} |x_{S} ) = \prod\limits_{s \in S} {\frac{1}{{\sqrt {2\pi } \sigma_{m} }}} } \exp \left[ { - \frac{{\left( {y_{s} - \mu_{m} } \right)^{2} }}{{2\sigma_{m}^{2} }}} \right].$$

The parameters $\mu_{m}$ and $\sigma_{m}$ are the mean and variance of the $m$th target region, respectively. In our experiment, $m$ is set to 2, which is consistent with the literature [19].

Substituting (5) and (7) into (4), and taking logarithms on both sides of (4), we can get

8 $$\begin{aligned} \hat{X} & = \arg \mathop { \hbox{max} }\limits_{x} (\ln P(X) + \ln P(Y|X)) \\ {\kern 1pt} & = \arg \mathop {\hbox{max} }\limits_{x} \left\{ { - \sum\limits_{c \in C} {V_{\text{c}} (x_{s} )} - \left[ {\sum\limits_{s \in S} {\left( {\ln (\sqrt {2\pi } \sigma_{m} ) + \frac{{\left( {y_{s} - \mu_{m} } \right)^{2} }}{{2\sigma_{m}^{2} }}} \right)} } \right]} \right\} \\ & = \arg \mathop {\hbox{min} }\limits_{x} \left\{ {\sum\limits_{c \in C} {V_{\text{c}} (x_{s} )} + \left[ {\sum\limits_{s \in S} {\left( {\ln (\sqrt {2\pi } \sigma_{m} ) + \frac{{\left( {y_{s} - \mu_{m} } \right)^{2} }}{{2\sigma_{m}^{2} }}} \right)} } \right]} \right\} \\ & = \arg \mathop {\hbox{min} }\limits_{x} \left( {U_{1} (X,Y) + U_{2} (X)} \right) \\ \end{aligned}$$

where $U_{1} (X,Y) = \ln P(Y|X) = \sum\limits_{s \in S} {\left( {\ln (\sqrt {2\pi } \sigma_{m} ) + \frac{{\left( {y_{s} - \mu_{m} } \right)^{2} }}{{2\sigma_{m}^{2} }}} \right)}$, $U_{2} (X) = \sum\limits_{c \in C} {V_{\text{c}} (x_{s} )}$; $U_{1} (X,Y)$ is conditional energy function (i.e., feature field energy), $U_{2} (X)$ is prior energy function (i.e., labeling field energy), $U(X,Y) = U_{1} (X,Y) + U_{2} (X)$ is MRF energy.

In summary, the calculation steps of MRF energy can be obtained as follows:

1. K-Means algorithm is used to initial segmentation, where *K* = 3, we divide the lung parenchyma, lung nodules and other lung tissue (blood vessels, pleura and highlight tissue, etc.) into three categories;
2. Solving the parameters (mean $\mu_{m}$ and variance $\sigma_{m}$) of the Gaussian mixture model using the expectation maximization method;
3. The total MRF energy $U(X,Y)$ is obtained from Eq. (8).

The MRF energy $U(X,Y)$ of each pixel can be calculated by (8). For the convenience of distinguishing and discussing later, the $U(X,Y)$ energy matrix is flattened into one-dimensional vector $u(x)$, where $1 \le x \le M*N$ represents the coordinate position of each pixel. MRF energy instead of intensity information is used for constructing region term of active contour model [31]; the region term can be constructed as follows:

9 $$E_{R} (\phi ) { = }\sum\limits_{i = 1,2} {\int_{\varOmega } {\left( {u(x) - f_{i} (x)} \right)^{2} M_{i} (\phi )} {\text{d}}x}$$

$f_{1} (x )$ and $f_{2} (x )$ are the mean values of MRF energy inside and outside of the contour curve, respectively. $M_{1} (\phi ) = H(\phi )$ represents the inside of the curve, and $M_{2} (\phi ) = 1 - H(\phi )$ represents the outside of the curve, where $H(\phi )$ is Heaviside function, $H(\phi ) = \frac{1}{2}\left( {1 + \frac{2}{\pi }\arctan \frac{\phi }{\varepsilon }} \right)$.

**Fig. 7:**
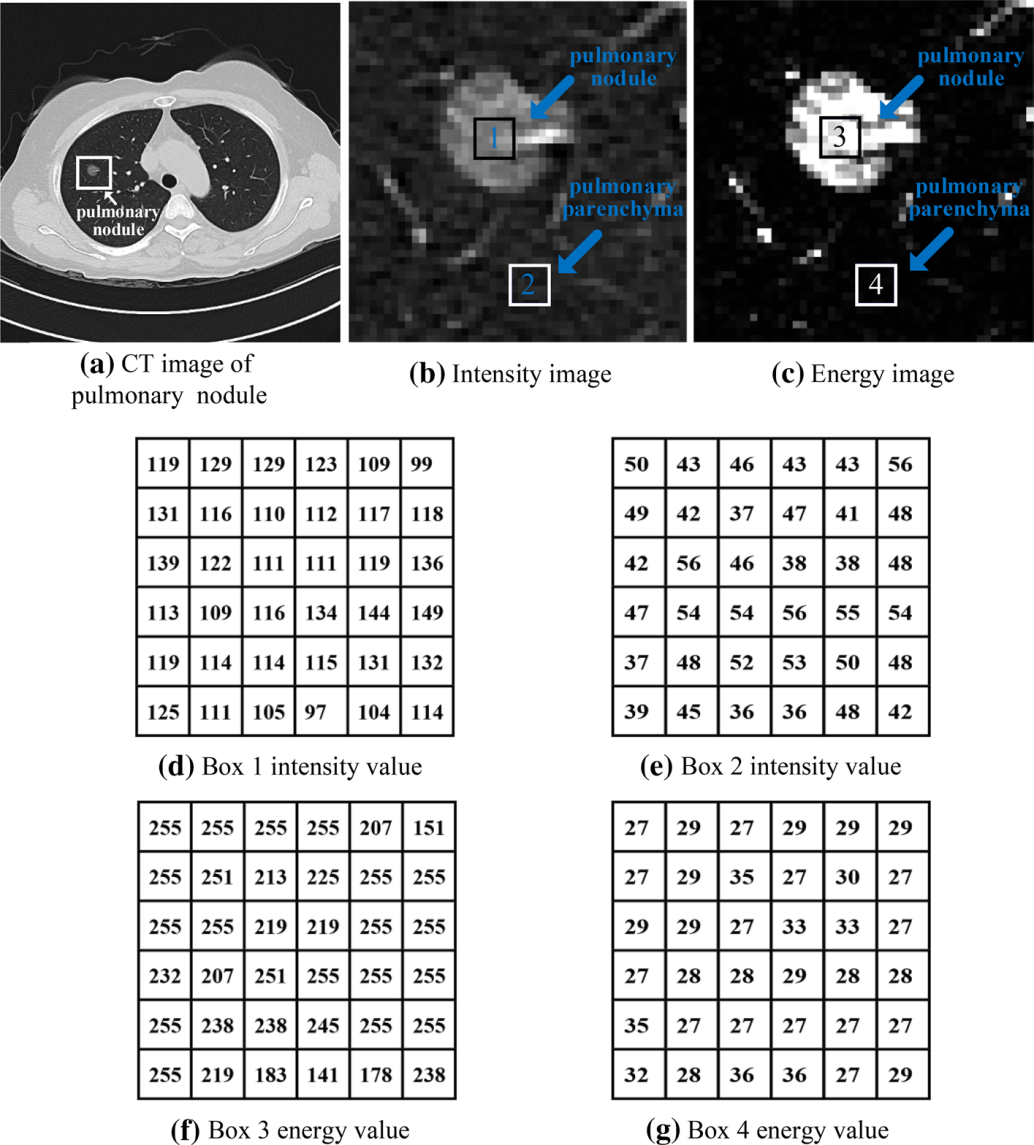
Contrast enhancement by MRF energy

It can be seen intuitively from Fig. 7 that MRF energy can significantly enhance the contrast between small GGO pulmonary nodule and background region. To further confirm the contrast enhancement effect, the intensity values and MRF energy values of pulmonary nodule and background region were compared and analyzed. Figure 7a is a CT image of a patient, the region drawn in the box is where the pulmonary nodule is located at. Figure 7b, c shows the intensity image and energy image of the region that is drawn in the box, respectively. Box 1 and 2 in Fig. 7b represents the pulmonary nodule region (segmented region) and the pulmonary parenchyma region (background region), respectively. Box 3 and 4 in Fig. 7c is similar. Figure 7d, e shows the intensity values corresponding to box 1 and 2, respectively, and Fig. 7f, g shows the energy values corresponding to box 3 and 4, respectively. Comparing the intensity value of box 1 (Fig. 7d) and the energy values of box 3 (Fig. 7e), it can be found that the energy values of the pulmonary nodule are significantly increased. Comparing the intensity values of box 2 (Fig. 7f) and the energy values of box 4 (Fig. 7g), it can be found that the energy values of the pulmonary parenchyma are reduced. Therefore, MRF energy can significantly enhance the contrast between pulmonary nodule and pulmonary parenchyma. In addition, the conditional probability $P(Y|X)$ is selected as the Gaussian mixture distribution when modeling the feature field of MRF. It effectively utilizes the local statistical information, which can reduce the suspicious boundary caused by factors such as intensity inhomogeneity and noise.

### Construction of the boundary detection term based on Bayesian probability difference

Assuming that the intensity of image pixels obeys the Gaussian mixture model (GMM) distribution [32], the specific form is a linear combination of Gaussian distributions:

10 $$p(x) = \sum\limits_{k = 1}^{K} {\alpha_{k} } N(x|\mu_{k} ,\varSigma_{k} )$$

where $\mu_{k}$ and $\varSigma_{k}$ represent the mean and variance of the $k$th Gaussian distribution, respectively. $\alpha_{k}$ represents the coefficients of the Gaussian mixture term, and $0 \le \alpha_{k} \le 1$, $\sum\limits_{k = 1}^{K} {\alpha_{k} } = 1$.

$X = \left\{ {x_{1} ,x_{2} , \ldots ,x_{n} } \right\}$ denotes a dataset consisting of $n$ pixels of an image, $Z = \left\{ {z_{1} ,z_{2} , \ldots ,z_{n} } \right\}$ is a dataset consisting of $n$ implicit data (i.e., implicit variables), and $\theta = \left\{ {\alpha_{k} ,\mu_{k} ,\varSigma_{k} |k = 1,2, \ldots ,K} \right\}$ is the parameters of Gaussian mixture distribution. The optimal parameter $\theta$ can be obtained by solving the maximum of logarithmic likelihood function. The likelihood function is as follows:

11 $$L(\theta ) = \ln p(X|\theta ) = \ln \sum\limits_{z} {p(X,Z|\theta )} .$$

The above formula can be solved effectively by EM algorithm [33], and then the optimal parameter set $\theta$ can be obtained. Further, $p_{k} (x_{i} |\theta_{k} )$ can be obtained by $\theta$, which represents the $k$th Gaussian component. For sample $x_{i}$, $p(k|x_{i} ,\theta )$ is the posterior probability of the $k$ th Gaussian component, which is defined as follows:

12 $$p(k|x_{i} ,\theta ) = \frac{{\alpha_{k} p_{k} (x_{i} |\theta_{k} )}}{{\sum\limits_{k = 1}^{K} {\alpha_{k} p_{k} (x_{i} |\theta_{k} )} }}.$$

Therefore, for any pixel $x$, the Bayesian posterior probability of the pixel $x$ that belonging to the pulmonary nodule class can be calculated, denoted as $p ( 1|x,\theta )$. Similarly, the Bayesian posterior probability of the pixel $x$ that belonging to the background region class can be calculated, denoted as $p(2|x,\theta )$. Thus, the boundary stopping function based on Bayesian probability difference can be obtained as follows:

13 $$S = \left| {p(1|x,\theta ) - p(2|x,\theta )} \right|.$$

According to the edge-based active contour model [34], the boundary detection terms can be constructed as follows:

14 $$E_{E} ( {\text{C)}} = \oint_{C} {g\left( {\left| {\nabla I\left( {C\left( s \right)} \right)} \right|} \right){\text{d}}s} { = }\oint_{C} {S{\text{d}}s} = \int_{\varOmega } {S\delta \left( \phi \right)\left| {\nabla \phi } \right|{\text{d}}x} .$$

**Fig. 8:**
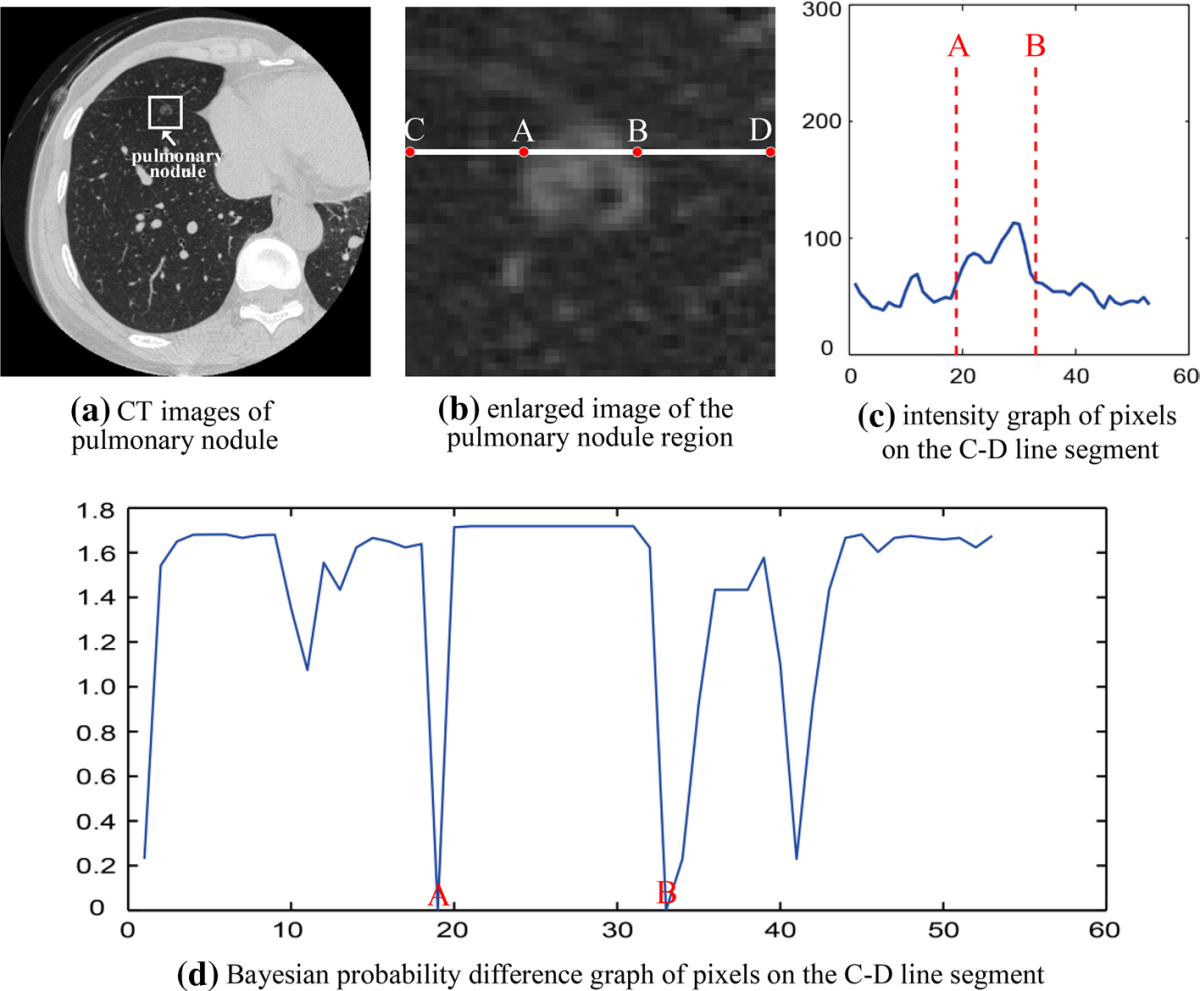
Boundary detection based on Bayesian probability difference

Figure 8 is an effect diagram of the boundary detection function based on Bayesian probability difference. Figure 8a is a CT image of a patient, the region drawn in the box is where the pulmonary nodule is located at, and the pulmonary nodule has a blurred boundary. Figure 8b is an enlarged image of the region drawn in the box, A–B line segment passes through the pulmonary nodule, point A is the left boundary, and B is the right boundary. The X and Y coordinates of points A and B in the image are (20, 19) and (20, 33), respectively. Figure 8c is an intensity graph of pixels on the C–D line segment. Figure 8d is a Bayesian probability difference graph of pixels on the C–D line segment. As we can be seen from Fig. 8, the Bayesian probability difference on points A and B is 0, so the probability difference can effectively detect the blurred boundary of the pulmonary nodule. The intensity values of points A and B did not change significantly, so the gradient calculated by the intensity values would not change much. Therefore, the blurred boundary of the GGO pulmonary nodule cannot be effectively detected by the gradient information.

### Construction and solution of energy function

The final energy function of the integrated active contour model can be obtained by substituting (9) and (14) into (2), specifically as follows:

15 $$\begin{aligned} E (\phi )& = \lambda_{ 1} \int_{\varOmega } {\left( {u(x) - f_{1} (x)} \right)^{2} H(\phi )} {\text{d}}x \\ & \quad + \lambda_{ 2} \int_{\varOmega } {\left( {u(x) - f_{2} (x)} \right)^{2} \left( {1 - H(\phi )} \right)} {\text{d}}x \\ & \quad + \mu \int_{\varOmega } {S\delta \left( \phi \right)\left| {\nabla \phi } \right|{\text{d}}x} + \nu \int_{\varOmega } {\frac{1}{2}\left( {\left| {\nabla \phi } \right| - 1} \right)^{2} {\text{d}}x} . \\ \end{aligned}$$

At the right of (15), the first two terms are region term, the third one is boundary detection term, and the fourth one is the regular term which guarantees that the level set function $\phi$ is kept as the symbolic distance function [35], i.e., guaranteeing that $\left| {\nabla \phi } \right| = 1$. $u(x)$ is defined by (9), and ${\text{S}}$ is defined by (13). Next, we will solve the energy function.

First, level set function $\phi$ is fixed. Taking the partial derivative of the energy functional $E(\phi )$ with respect to $f_{1} (x)$ and $f_{2} (x)$, respectively, and making them equal to zero, we can get that

16 $$f_{1} (x) = \frac{{\int_{\varOmega } {u(x)H(\phi ){\text{d}}x} }}{{\int_{\varOmega } {H(\phi ){\text{d}}x} }},\quad f_{2} (x) = \frac{{\int_{\varOmega } {u(x)\left( {1 - H(\phi )} \right){\text{d}}x} }}{{\int_{\varOmega } {\left( {1 - H(\phi )} \right){\text{d}}x} }}.$$

Second, $f_{1} (x)$ and $f_{2} (x)$ are fixed. Taking the derivative of the energy functional $E(\phi )$ with respect to $\phi$, $\phi$ is solved by gradient descending flow, details as follows:

17 $$\begin{aligned} \frac{\partial \phi }{\partial t} & = \lambda \cdot \delta (\phi )\left( {\left( {u\left( x \right) - f_{2} \left( x \right)} \right)^{2} - \left( {u\left( x \right) - f_{1} \left( x \right)} \right)^{2} } \right) \\ & \quad + \mu \cdot \delta (\phi )div\left( {S\frac{\nabla \phi }{|\nabla \phi |}} \right){ + }\nu \cdot \left( {\nabla^{2} \phi - div\left( {\frac{\nabla \phi }{|\nabla \phi |}} \right)} \right) \\ \end{aligned}$$

where $\delta (\phi ) = \frac{\varepsilon }{\pi }\frac{1}{{\varepsilon^{2} + \phi^{2} }}$ is the derivative of $H(\phi )$, also known as the Dirac Delta function.

## Figures and Tables

**Table 4:**
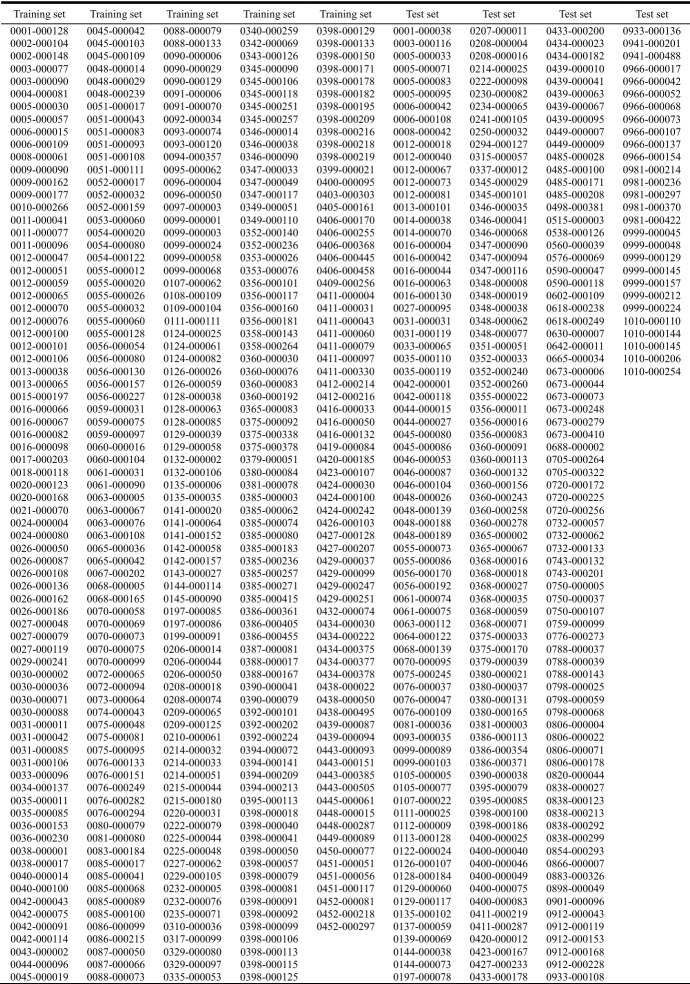
ID numbers of LIDC-IDRI data

## Data Availability

The datasets used and/or analyzed during the current study are available from the corresponding author on reasonable request.

## Appendix

Table 4 gives the ID numbers of all data in LIDC-IDRI. The meaning of the numerical sequence in the table has been explained in the experimental data description section.

## Abbreviations

CAD: Computer-aided diagnosis
GGO: Ground glass opacity
IACM_MRFEBPD: Integrated active contour model based on Markov random field energy and Bayesian probability difference
MRF: Markov random field
CT: Computed tomography
EM: Expectation maximization
LIDC-IDRI: Lung image database consortium and image database resource initiative
IOU: Intersection over union
ID: Identifier
GMM: Gaussian mixture model
LGDF: Local Gaussian distribution fitting energy
SFCM_LCM: Spatial fuzzy clustering with level set methods
LRBAC: Localizing region-based active contours
MRF_SA: Markov random field with simulated annealing algorithm
